# Effect of Process Parameters on the Crystallinity and Geometric Quality of Injection Molded Polymer Gears and the Resulting Stress State during Gear Operation

**DOI:** 10.3390/polym15204118

**Published:** 2023-10-17

**Authors:** Damijan Zorko

**Affiliations:** Faculty of Mechanical Engineering, University of Ljubljana, Aškerčeva 6, 1000 Ljubljana, Slovenia; damijan.zorko@fs.uni-lj.si

**Keywords:** injection molding, polymer gears, gear quality, stress, crystallinity

## Abstract

The quality of gear manufacturing significantly influences the way load is distributed in meshing gears. Despite this being well-known from practical experience, gear quality effects were never systematically characterized for polymer gears in a manner able to account for them in a standard calculation process. The present study employs a novel combination of numerical and experimental methods, leading to a successful determination of these effects. The findings of the study enhance existing gear design models and contribute to a more optimized polymer gear design. The study first explores the effect of injection-molding parameters on the gear quality and secondly the effect of resulting gear quality on the stress conditions in a polymer gear pair. For the gear sample production, different combinations of process parameters were investigated, and a classic injection-molding and the Variotherm process were utilized. Gear quality and crystallinity measurements were conducted for all produced gears, providing insights into the correlation between them. Based on the evaluated gear quality of produced samples, the effect of gear quality was further studied by numerical means within a meaningful range of quality grades and transmitted loads. Special attention was dedicated to lead and pitch deviations, which were found to exert a noteworthy influence on the stress state (both root and flank) of the gear. The effect of lead deviation was most pronounced when improving the gear quality from grade Q12 to grade Q10 (30% to 80% stress reduction, depending on the load). However, enhancing the quality grade from Q10 to Q8 yielded less improvement (5% to 20% stress reduction, depending on the load). A similar pattern was evident also for pitch deviations.

## 1. Introduction

High-performance polymer gears represent a contemporary technology that is progressively supplanting traditional metal gears due to an array of benefits. Apart from the evident advantage of substantial mass reduction, these gears can operate effectively without the need for supplementary lubrication, rendering them particularly appealing for scenarios where lubricants are undesirable, such as in printers, household appliances, and medical equipment. Notably, polymer gears exhibit superior vibration-dampening capabilities and significantly reduced operational noise [1]. The inherent corrosion resistance and resilience to chemical influences commonly observed in polymers also enable these gears to function effectively in environments where corrosive agents are present. Another highly significant advantage lies in their cost-efficient mass production through injection molding processes. Engineering polymers from the polyoxymethylene (POM) and polyamide (PA) families are usually employed for polymer gear applications, due to the good mechanical [2], tribological [3], and processing properties [2] these materials exhibit. Further performance enhancements can be achieved by introducing reinforcing fibers and internal lubricants [4], or by going up the polymer pyramid and employing materials from the Polyether-ether-ketone (PEEK) family [5].

Polymer gears can fail due to different failure modes, i.e., fatigue (root or flank), wear, or thermal overload, which results in severe plastic deformation. The majority of available studies focus on the known failure modes in order to improve the gear performance or provide comprehensive design methods that would enable a more optimized gear design. The VDI 2736 [6], published in 2014, provides gear design guidelines and analytical models to control all the recognized failure modes. While the guidelines serve as a good basis, over the years, several points for improvement have been recognized. The guidelines employ the same root and flank strength control models as the ones used in the DIN 3990 [7] standard, which is used for steel gears. Polymer materials are much more flexible than steel ones, and plastic teeth exhibit higher teeth deflection while meshing, leading to an increase in the contact ratio [8]. The use of polymer gears is limited also by a major lack of available material data, which are crucial for gear design. In order to conduct reliable root strength control, the information on the material’s fatigue strength, in the form of a stress vs. number of load cycles curve, is required. Similarly, when conducting wear control, the wear factors for the material pair of choice are required. Generating the essential material data requires specialized test equipment, experience, and much time. To speed up the generation of these data, variations of accelerated methodologies for polymer gear testing were presented in several studies [9,10], enabling the determination of the load-bearing characteristics of a gear pair. Such methods are also well-matched for evaluating the appropriateness of the chosen material combination. The accelerated gear testing techniques underwent further enhancements in the study conducted by Lu et al. [11], in which they derived an equivalent S-N curve through the Locati approach. In comparison to the conventional steady loading approach, the implementation of accelerated life testing led to a 45% reduction in the testing period, accompanied by an average error rate of 10.64%. Based on the recognized polymer gear failure modes and available design methods, a comprehensive polymer gears design optimization approach was introduced in [12]. Attempts have been undertaken to integrate machine learning algorithms into the gear design [13,14], demonstrating their effectiveness in assessing unconventional gear designs.

Wear has been another widely studied topic for polymer gears. The involute, or any other recognized tooth shape [15,16,17,18], which complies with the law of gearing, enables a steady and smooth power transmission. Deviating from the ideal tooth shape, either by wear or reduced meshing stiffness [19], leads to transmission error and increased noise, vibrations, and harshness (NVH). It was found that employing a suitable material combination for the drive and the driven gear, e.g., a combination of POM and PA gears, results in improved wear performance [20,21]. Different wear measuring methods are possible to determine the wear factors for polymer gears; the most commonly used are the gravimetric method [22], the tooth thickness-reduction method [22], and the image processing method [23]. Also, advanced in-situ wear measuring methods can be employed [24]. The study conducted by Černe [24] introduces an optical methodology for experimental analysis, involving the assessment of in-mesh gear tooth deflection via high-speed camera recordings. This technique encompassed two distinct image processing methods: the established digital image correlation method and an innovative edge displacement detection method. The freshly devised edge displacement detection method harbors considerable potential for enhancing experimental analysis focused on protracted wear and the accumulation of strain over time.

The sliding and rolling motion between the meshing flanks results in gears heating up during operation. For plastic materials, some portion of the heat generation is also due to hysteretic effects; however, this portion was identified as rather small [25]. Recent efforts have yielded various models for predicting the operational temperature of polymer gears [26,27,28]. In order to improve the thermal performance of polymer gears, different concepts were introduced, either by modifying the tooth width in the most loaded tooth area [29], introducing additional holes in the gear body in order to facilitate the convective heat transfer to the surroundings [30], or employing a hybrid polymer gear concept [31,32] where the metal inserts improve the heat conduction. Okubo et al. [33] monitored structural transformations within a polyamide PA 66 gear during operation, leveraging ex-situ Raman and Fourier-transform infrared spectroscopy. Their investigation unveiled shifts in crystallinity, the breakage of amide-related bonds, the occurrence of a trans-gauche (TG) transformation, and alterations in the inner stress state of the PA 66 gear over time. The conclusion drawn was that these changes interplay during gear operation and are correlated with the failure of the PA 66 gear.

While polymer gears offer numerous benefits, they also come with certain drawbacks. Among the notable shortcomings are diminished load-bearing capacity, inferior thermal conductivity, temperature stability, and manufacturing precision. Of particular significance is the load-bearing capacity, prompting a range of investigations aimed at enhancing this attribute through avenues like optimized gear design [12,34] or enhanced materials [23]. However, there is a notable scarcity of comprehensive inquiries tackling the geometric precision of injection-molded polymer gears. This is evident in the limited number of studies devoted to this subject.

The majority of polymer gears produced in mass quantities are manufactured using the injection-molding process. When utilizing this method of production, it becomes imperative to account for the shrinkage and warping that occurs during the cooling phase of the material [35,36]. To ensure the gears attain a desirable level of quality, due attention must be paid to aspects like tool design, tool production [37], and process parameters. Advanced simulation tools can predict shrinkage and warping, effectively capturing the material’s behavior with a commendable level of accuracy. During the initial stages of tool design, adjustments are made to the mold to align with the projected outcomes from simulations. ISO 1328 [38,39] and DIN 3961/62 [40] define thirteen quality grades Q, ranging from the best grade Q0 to the worst Q12. In routine polymer gear production, the achieved quality grades typically range between quality grades Q10 and Q12. Elevating precision to higher quality grades within the Q8 range necessitates meticulous process control.

The extent of shrinkage is primarily contingent on both the utilized material and the parameters of the manufacturing process. Traditionally, the evaluation of gear quality within industrial settings has primarily relied on coordinate measuring machines (CMMs) [41,42] or double-flank rolling test devices [43,44]. These techniques provide dependable means for assessing the requisite geometric parameters that underpin overall gear quality. Nevertheless, recent years have witnessed significant efforts in applying optical methodologies like laser measurements and structured light 3D scanning. These innovations aim to expedite, enhance accuracy, and offer comprehensive gear inspection capabilities. Urbas et al. [45] directed their attention to the utilization of structured light 3D optical scanning to gauge the gear quality of polymer gears. They conducted a comparative analysis between the outcomes derived from CMM measurements and those obtained through 3D scanning. The results demonstrated that key parameters essential for assessing gear quality could be reliably evaluated using the 3D scanning technique. However, for parameter identification based on correlation with a theoretical CAD gear model [46], it’s imperative to employ a 3D scanner with adequate precision and also implement a suitable 3D scan alignment approach. Moreover, optical measurement methods open avenues for comprehensive 3D assessments of the overall surface geometry of the gearing.

The majority of materials used for polymer gears are from the semi-crystalline family. When molding the gears, the solidification of the material first begins at the contact of the melt with the mold. Due to the rapid cooling, too little time is available for the formation of crystalline structures, so the surface of injection-molded gears is usually of an amorphous structure, which is usually associated with poorer mechanical properties. The structure of the material can be improved by proper tool tempering [47]. At higher cooling rates, the degree of crystallinity will be lower, and vice versa. In the manufacture of polymer gears, what levels of accuracy are achievable with each manufacturing technology and the structure of the material are important, as this has an impact on the service life of polymer gears. This article provides a comprehensive study on the effect of injection-molding process parameters on the relation between crystallinity and geometric quality of polymer gears.

Furthermore, the study seeks to examine how the manufacturing quality of polymer gears affects their mechanical performance during operation, and consequently, their overall lifespan. Conducting this investigation solely through experimental means would be a daunting task due to the significant challenges involved in producing gears with predetermined quality grades. It is even more challenging to create gears with specific quality parameters, such as lead profile and pitch, in selected predefined quality grades. Therefore, a novel approach that combines numerical and experimental methods was employed, leading to the successful determination of these effects. To the best of the authors’ knowledge, there has not been a systematic study that delves into these gear quality effects and characterizes them comprehensively. The discoveries from this research enhance existing gear design models and contribute to the development of more optimized designs for polymer gears.

## 2. Materials and Methods

A systematic combination of numerical and experimental methods was employed to study the effects of tool design and process parameters on the geometric quality of injection-molded polymer gears. Additionally, the correlations between the crystallinity and gear quality were investigated. Finally, the effect of gear quality on the stress state in the gears is evaluated.

### 2.1. Materials

Gear samples were injection-molded using the commercially available Delrin 100 NC010 material granulate (DuPont, Wilmington, DE, USA). The material of choice is a high-viscosity homopolymer (POM-H) and is in practice very commonly used for polymer gears as it provides good fatigue strength and tribological behavior in terms of low wear and friction. The employed material is also very suitable for injection molding as it does not require high processing temperatures and is easy to fill molds. The processing parameters proposed by the material’s manufacturer are provided in Table 1, and the basic properties of the employed material are summarized in Table 2.

### 2.2. Sample Preparation

**Figure 1:**
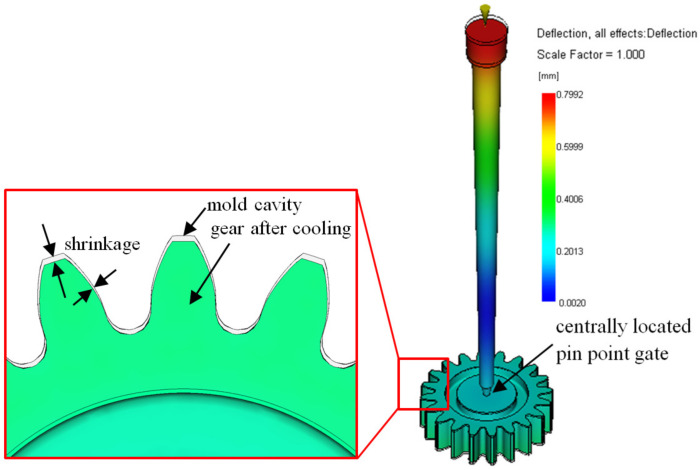
Simulated shrinkage of the molded gear and gate after cooling.

A dedicated injection-molding tool with a standard frame and an exchangeable single-cavity mold insert was produced. Prior to the production of the mold cavity, injection-molding simulations were conducted in Autodesk Moldflow Synergy 2015 (Autodesk, Inc., San Francisco, CA, USA) in order to verify the cavity filling and the gate design, and to calculate the shrinkage of the gear after cooling (Figure 1). The simulation-calculated shrinkage of 2% was considered when producing the cavity by the wire electrical discharge machining (WEDM). The melt was delivered to the cavity through a standard PGH4A5-52.5-P1.3-A1-K20-B30 Misumi pinpoint gate insert. The gate location was positioned in the center of the gear, as presented in Figure 1. The mold temperature was measured with a thermocouple type J (Fe-CuNi DIN EN60584) on the injection and the ejection side of the tool. A hot runner system (Mold-Masters Europa GmbH, Baden-Baden, Germany) with a single nozzle was used.

Prior to injection molding the samples, the material granulate was dried and dehumidified. The drying temperature was set to 80 °C and the drying time was 3 h. The moisture content after the drying process was below 0.1%. After drying, the sample gears were injection-molded on a KraussMaffei CX80-160 (KraussMaffei Technologies GmbH, Vaterstetten, Germany) injection-molding machine. Several combinations of process parameters, within a feasible range, were employed in order to study the effect of process parameters on the gear quality (and the degree of crystallinity). Additionally, a classic injection-molding process and Variotherm [52] technology were used for the production of test samples. The employed combinations of process parameters are presented in Table 3. For each selected parameter set, gear samples were molded until the steady state conditions were achieved on the molding machine. After that, 30 gear samples were molded for each parameter set, which were then subject to further studies, i.e., geometric quality and crystallinity measurements.

**Figure 2:**
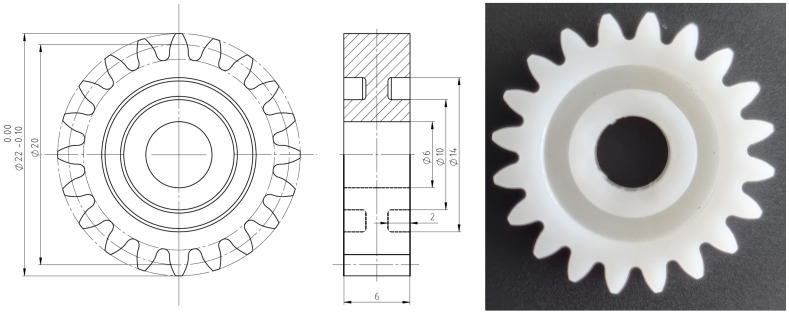
The specified gear geometry, and the injection-molded gear.

The produced gear geometry was in line with the test gear geometry employed in several authors’ previous studies [53,54]. The main gear geometric parameters are presented in Table 4. The gear body was optimized for the injection-molding process, considering the melt flow, wall thickness, and symmetrical filling of the gear ring (Figure 2). Gears were initially molded without the center hole, which was later additionally machined on a precision CNC milling machine.

### 2.3. Crystallinity Measurement

When injection molding, the tendency is usually to achieve a relative crystallinity degree as high as possible while also taking into account the efficiency of the molding process. A higher degree of crystallinity leads to reduced internal stress in the molded part, increases its strength, stiffness, and heat resistance, and has a beneficial effect on geometric stability.

**Figure 3:**
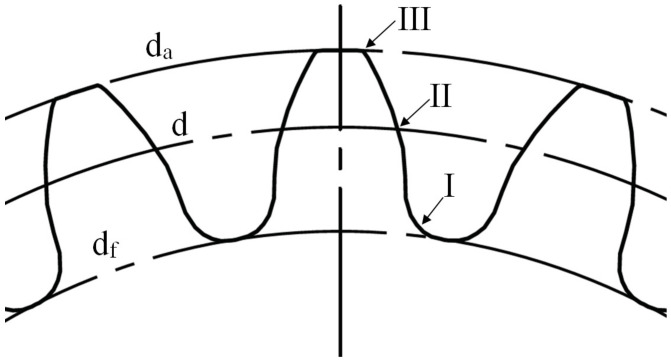
Region I (at the root diameter d_f_), region II (at the reference diameter d) and region III (at the tip diameter d_a_), along the tooth height, were selected for the crystallinity measurement.

For each set of parameters presented in Table 2, three gears were randomly selected and subject to crystallinity measurements by employing the Flash DSC method on the Mettler Toledo Flash DSC 1 apparatus (Mettler Toledo Inc., Greifensee, Switzerland). On each selected gear, a tooth was again randomly chosen, and samples of appropriate size, ranging between 10 ng and 1000 ng, were cut from each analyzed gear. The samples were cut from three regions along the tooth height, as presented in Figure 3. For comparison, the neat resin was also analyzed by the Flash DSC method, and the crystallinity curves were generated.

Differential scanning calorimetry (DSC) can be used to track the thermal transitions of polymer materials and the associated enthalpies and crystallization of polymers. The Flash DSC method employs the same underlying principle; however, it allows very high rates of heating (up to 2.4 × 10^6^ °C/min) and cooling (up to 2.4 × 10^5^ °C/min) of the material’s sample. Using this method, the temperature conditions to which the material is exposed during the injection-molding cycle can be simulated, and the process can be optimized. The scan temperature range for the conducted Flash DSC measurements was from 60 °C to 260 °C.

In addition to optimizing the injection-molding process, Flash DSC can be also used to study the crystallization kinetics of thermoplastic materials, study their morphology, and determine the material’s minimum cooling rate during processing. It is important to have knowledge on the mentioned phenomena in order to prevent the product crystallizing during use, which in many cases leads to failure. The method can be also used to prove that the material has been processed under inappropriate conditions, which is often the cause of product failure, similar to cold crystallization. The effects of the material’s crystallinity degree on the glass transition temperature can also be determined using the Flash DSC method for partially crystalline thermoplastics, and it is possible to trace the degradation of amorphous thermoplastics with respect to the relaxation enthalpy at the glass transition, which occurs at higher heating rates in the case of degraded material.

### 2.4. Gear Quality Measurements

**Figure 4:**
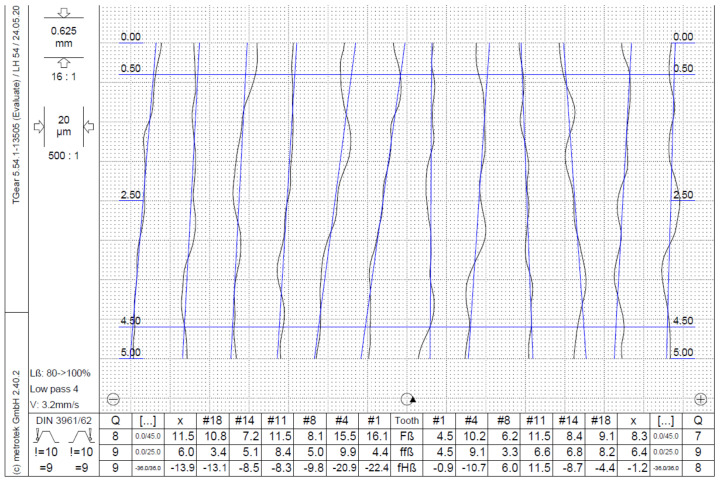
A representative example of the lead quality measurements report. Six teeth were measured and the lead quality parameters, i.e., Total helix deviation—$F_{\beta }$, Helix form deviation—$ff_{\beta }$ and Helix slope deviation—$fH_{\beta }$, were evaluated for the right and left tooth flank. Q denotes the achieved quality grade, x is the average value of six measurements, # denotes the number of the measured tooth, ! defines the requested quality grade.

After manufacturing, the gear sample’s geometric quality gears were measured on the LH54 (Wenzel Messtechnik GmbH, Blaubeuren, Germany) CMM gear-measuring machine. The gear quality was assessed according to DIN 3961/62. Gears were measured in a controlled environment; ambient temperature was 23 +/−1 °C and relative humidity was 50 +/−5%. The standardized CMM measurements were taken for six teeth on each gear and for a selected rating section on the chosen teeth. Both flanks of each measured tooth were evaluated (Figure 4). Three gears made from the series with fixed parameters were measured, and the worst measured results are presented in this study.

### 2.5. Stress Evaluation

The effect of gear production quality on the stress evolution in the gear is adequately accounted for in the design standards applicable to steel gears. In both the DIN 3990 [7] and ISO 6336 [56], the influence of gear manufacturing quality is considered through factors $K_{F\beta }$ and $K_{F\alpha }$ when determining the root stress. The DIN 3990 (Method C) equation is also used for root stress calculation in the VDI 2736 [6] guideline, and it reads as:

(1) $$\sigma_{F}=K_{A}\cdot K_{v}\cdot K_{F\beta }\cdot K_{F\alpha }\cdot Y_{Fa}\cdot Y_{Sa}\cdot Y_{\varepsilon }\cdot Y_{\beta }\cdot \frac{F_{t}}{b\cdot m}$$

where $\sigma_{F}$ is the root stress in the gear, $K_{A}$ is the application factor, taking into account the externally influenced variations of input or output torque, $K_{V}$ is the dynamic factor, which considers the effect of internal dynamic effects, and $K_{F\beta },\;K_{F\alpha }$ are the face load and transverse load factors for the tooth root stress. $K_{F\beta }$ and $K_{F\alpha }$ account for the effects on the root stress, which result from uneven load distribution over the face width and in the transverse direction. These factors are gear-profile independent and can be employed for arbitrary gear profiles. The considered gear profile geometry is further accounted for by the form factor $Y_{Fa}$, stress correction factor $Y_{Sa}$, contact ratio factor for root stress $Y_{\varepsilon }$, and helix angle factor for root stress $Y_{\beta }$.

In a similar manner, the VDI 2736 [6] equation for calculating the contact pressure reads as:

(2) $$\sigma_{H0}=Z_{H}\cdot Z_{E}\cdot Z_{\varepsilon }\cdot Z_{\beta }\cdot \sqrt{\frac{F_{t}}{b\cdot d_{1}}\cdot \frac{u+1}{u}\cdot K_{A}\cdot K_{V}\cdot K_{H\beta }\cdot K_{H\alpha }}$$

where $K_{H\beta }$ and $K_{H\alpha }$ are the face load and transverse load factors for the contact pressure. They account for the effects on the flank pressure, which result from uneven load distribution over the face width and in the transverse direction. The effect of the considered gear profile and material combination is further accounted for by the zone factor $Z_{H}$, elasticity factor $Z_{E}$, contact ratio factor for flank pressure $Z_{\varepsilon }$ and helix angle factor for flank pressure $Z_{\beta }$.

Apart from the geometric irregularities present in gears, factors $K_{F\beta }$, $K_{F\alpha }$, $K_{H\beta }$ and $K_{H\alpha }$ also encompass the deflections of the shafts, bearings, and housing, thermal expansion, bearing clearance, running-in effects, and micro-geometry adjustments.

**Figure 5:**
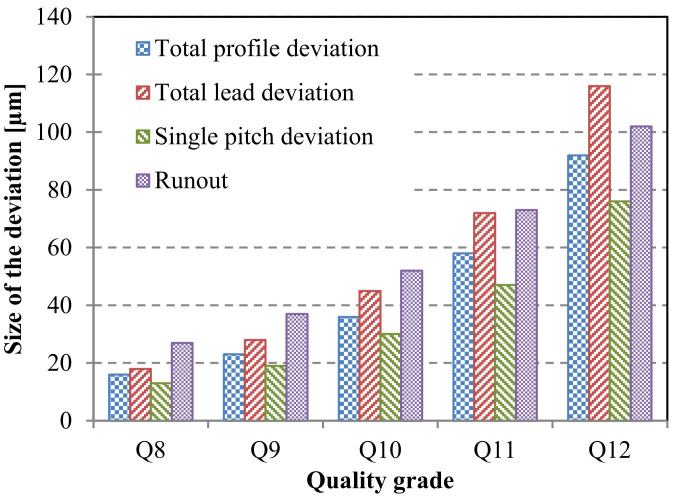
Limit size of the deviations for a selected gear quality grade (the borderline values defined according to DIN 3961/62).

When assessing the strength of polymer gears, the current VDI 2736 [6] design guideline lacks methods to account for these influences. Instead, there exists an empirical guideline that suggests, in cases where b/m≤12, the root load factor $K_{F}=K_{A}\cdot K_{V}\cdot K_{F\beta }\cdot K_{F\alpha }\approx 1\ldots 1.25$, and a similar guideline applies to the flank load factor $K_{H}=K_{A}\cdot K_{V}\cdot K_{H\beta }\cdot K_{H\alpha }\approx 1\ldots 1.25$. This assumption is being tested in this investigation, which involves the examination of gears with a b/m ratio of 6 and quality grades ranging from Q8 to Q12 (DIN 3961/62 [40]). The highest deviations for the main gear-quality parameter and individual quality grades from Q8 to Q12 are illustrated in Figure 5. For the purposes of this study, it was presumed that lead deviation and pitch deviation are the most influential parameters affecting the stress state. Consequently, these two parameters were analyzed by employing numerical means utilizing the finite element method (FEM).

The evaluated scenarios assumed a worst-case alignment of polymer gears of identical quality grade, with a focus on POM/POM gear pairing. In FEM simulations, the material characteristics were represented as linearly elastic, employing the material properties outlined in the manufacturer’s datasheet, as presented in Table 2. The presumption of linear elastic behavior is commonly employed in polymer gear-design stress calculations, as the computed strains remain below the polymer material’s yield point [8,27]. It was affirmed by Černe et al. [57] that the assumption of linear elastic mechanical behavior provides a suitably accurate approximation of the material’s performance for practical thermo-mechanical modeling purposes in gear design applications.

#### 2.5.1. The Effect of Lead Quality (Distribution of Load across the Tooth’s Width)

To determine load distribution across the tooth width, both lead deviation and elastic deformation of the tooth were taken into account. However, the model did not incorporate manufacturing deviations or deformations of other gearbox components such as shafts, housing, and bearings. Several numerical models have been formulated to assess the impact of lead deviations and tooth elastic deformations.

**Figure 6:**
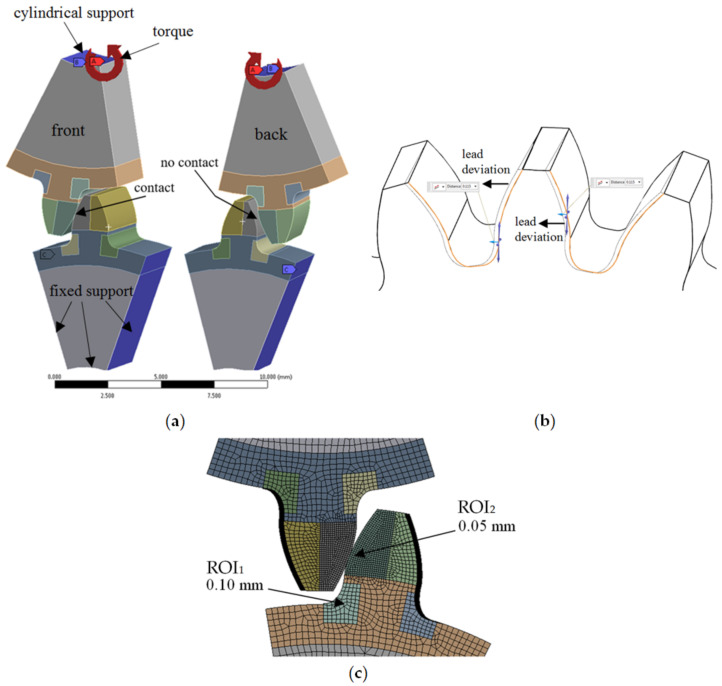
(**a**) Supports and loads in the numerical model employed for the lead-quality characterization (**b**) Principle of modeling the lead deviation, (**c**) Finite element mesh in the regions of interest ROI_1_ and ROI_2_, where the calculated stress was analyzed.

Given the complexity, the numerical modeling transpired in 3D. The geometric model encompassed a single-tooth segment of both the drive and the driven gear, situated at the highest point of single-tooth contact for the driven gear. The lead deviations for the assessed quality grades were depicted as demonstrated in Figure 6a. On the active side, the lead deviation extended away from the tooth (in the plus direction relative to the tooth thickness), while on the inactive side, it was oriented towards the tooth (in the minus direction). This deviation distribution was indicative of the worst-case scenario. Considering similar deviations on the coast side would augment tooth thickness and consequently bolster the root load-bearing capacity.

The gears were positioned into mesh to simulate the worst-case misalignment, aligning the maximum lead deviation of the drive gear with that of the driven gear. The mesh positioned the gears at the highest point of single-tooth contact for the driven gear, depicted in Figure 6b. Initial load resulted in a line contact on one side of the teeth due to the modeled lead deviations. The material’s elastic properties prompted the formation of a broader contact area under load, evident in the simulation outcomes.

To ensure robustness, a mesh-independence test was executed using the h-refinement technique, determining the suitable finite element size for subsequent simulations. This led to a region of interest (ROI_1_) in the root area with and element size of 0.1 mm and a region of interest (ROI_2_) on the flank, where the element size was 0.05 mm, depicted in Figure 6c. Quadratic SOLID186 and SOLID 187 elements were employed, maintaining an average element quality of 0.85. To simulate surface interactions, the analysis employed CONTA172 (for the drive gear) and TARGE169 (for the driven gear) elements. The establishment of contact interactions and frictionless behavior was facilitated through an augmented Lagrange method.

The stress obtained from the model accounting for lead deviations was contrasted with the stress computed for the identical gear pair featuring an ideal theoretical gear geometry. In order to measure the impact of lead deviation on root stress within the examined gear, a parameter denoted as $Q_{F\beta }$ was established. This parameter was determined by evaluating the ratio between the calculated stress:

(3) $$Q_{F\beta }=\frac{\sigma_{F,Q_{i}}}{\sigma_{F,ideal}}$$

in this context, $\sigma_{F,Q_{i}}$ represents the highest calculated root stress associated with the examined lead quality level, while $\sigma_{F,ideal}$ corresponds to the maximum root stress calculated for the gear pair featuring an idealized geometry. A comparable methodology was also applied to gauge the influence of lead deviation on contact pressure:

(4) $$Q_{H\beta }=\frac{\sigma_{H,Q_{i}}}{\sigma_{H,ideal}}$$

**Figure 7:**
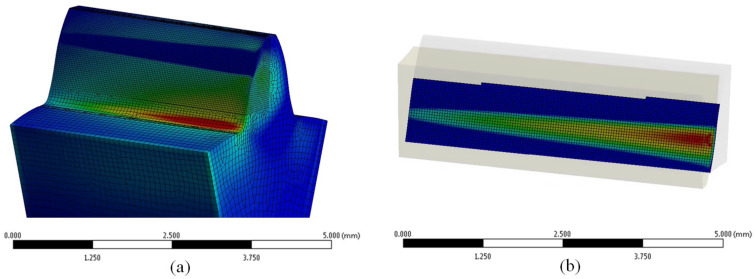
Simulation-calculated stress state, for a gear pair with lead deviations modeled in quality grade Q12, and loaded with 1.2 Nm torque: (**a**) The maximum principal stress distribution in ROI_1_, (**b**) The maximum contact pressure distribution in ROI_2_.

This entails introducing a novel parameter, denoted as $Q_{H\beta }$, which factors in the influence of lead quality on contact pressure. In this context, $\sigma_{H,Q_{i}}$ symbolizes the maximum contact pressure calculated for the examined lead quality grade, while $\sigma_{H,ideal}$ pertains to the peak contact pressure calculated for the gear pair characterized by an ideal geometry. In determining the maximum root stress, attention was directed towards the principal max-stress, as depicted in Figure 7a. Conversely, for the assessment of contact pressure, focus rested on the peak contact pressure amidst the contacting flanks, as illustrated in Figure 7b. Given the material’s elastic behavior, a range of different loads within a feasible span was scrutinized. Elevated torque levels yielded greater elastic deformation in the teeth, culminating in more effective load distribution across the tooth’s width. The simulations spanned load cases of $F_{T}/b=\left[6.67;13.33;\;20;\;26.67\right]$, with intermediate loads subject to linear interpolation.

#### 2.5.2. The Effect of Pitch Quality (LOAD Sharing among the Teeth)

**Figure 8:**
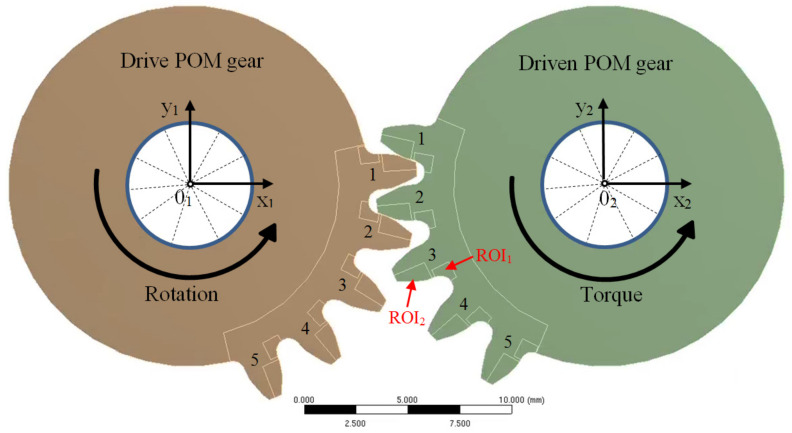
Gear pair geometry and boundary conditions of the numerical model used to study the effect of pitch deviation. Pitch deviations were modeled on the third (middle) tooth of the drive and driven gear.

**Figure 9:**
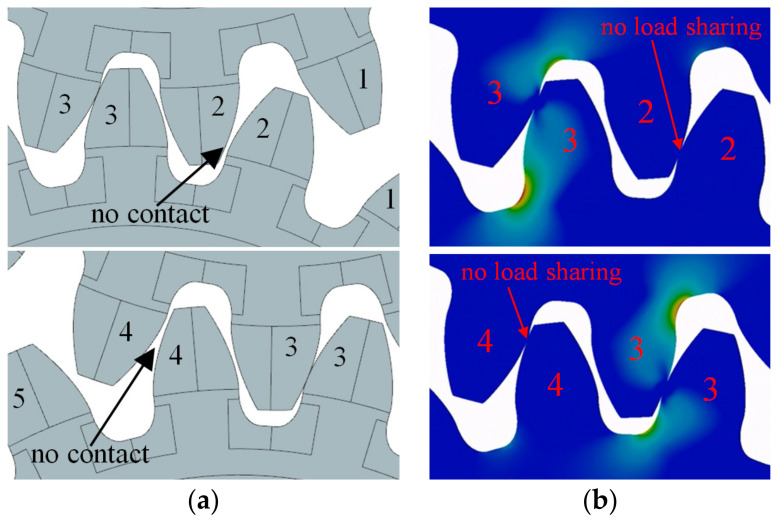
Effect of modeled pitch deviations (teeth pair 3) on the load sharing; when the teeth pair 3 is meshing there is no contact between teeth pair 2 and teeth pair 4: (**a**) Undeformed geometry/no load, (**b**) Stress distribution under load.

For exploring the influence of pitch deviation on load sharing between the teeth, a distinct numerical model was employed. This model was simplified into 2D, encompassing five teeth each for the drive and the driven gear (Figure 8). The middle tooth (tooth no. 3) on both the drive and driven gear was subjected to pitch deviations in a manner that induced the worst-case scenario. In terms of pitch deviations, the target tooth (tooth no. 3) exhibited a plus deviation on the active flank side and a minus deviation on the coast side. Figure 9a provides an overview of meshing for the un-deformed geometry, illustrating that contact is absent between teeth no. 2 before the pitch point, and similarly, there is no contact between teeth no. 4 after the pitch point. When under load, teeth deflect depending on the modeled size of pitch deviation, and the load sharing between teeth was altered or not even existent (Figure 9b).

The boundary conditions are depicted in Figure 8 for the numerical representation. The central hole of the drive gear was constrained at point 0_1_, positioned at the origin of the x_1_y_1_ coordinate system. Constraints were applied to translations along both the x_1_ and y_1_ directions, with unrestricted rotational movement around point 0_1_. Similarly, the driven gear was confined to point 0_2_, corresponding to the origin of the x_2_y_2_ coordinate system. For the drive gear, a roll angle of 55° around point 0_1_ was specified, whereas the driven gear encountered a torque countering the drive gear’s rotation. Subsequent analysis focused on the third tooth of the driven gear, which meshed through all the characteristic contact points.

Particular attention was given to two regions of interest (ROIs) within the tooth structure: the root region (ROI_1_) and the flank region (ROI_2_). To establish suitable element sizes for both ROIs, a mesh-convergence study utilizing the h-refinement method was undertaken. Consequently, the element size in ROI_1_ was fixed at 0.10 mm, while in ROI_2_, a value of 0.016 mm was determined.

Employing the outlined finite element method (FEM) model, the analysis factored in the elastic deformations of the teeth to assess the influence of pitch deviation. Taking into account the deflection induced by the applied load, contact was established between the second and fourth pairs of teeth as well. In scenarios involving substantial pitch deviations and lower loads, the deformation of the teeth was insufficient, resulting in the entire load being transferred through a single pair of teeth. The computed stress values for the gear-pair configuration with pitch deviations were juxtaposed with those calculated for the idealized geometry. Based on this comparison, the coefficient $Q_{F\alpha }$ was derived using the subsequent equation:

(5) $$Q_{F\alpha }=\frac{\sigma_{F,Q_{i}}}{\sigma_{F,ideal}}$$

where $\sigma_{F,Q_{i}}$ represents the maximum root stress calculated for the examined pitch-quality grade $Q_{i}$, while $\sigma_{F,ideal}$ corresponds to the maximum root stress computed for the gear pair with the ideal geometry. A similar approach was employed to ascertain the impact of pitch deviation on the contact pressure:

(6) $$Q_{H\alpha }=\frac{\sigma_{H,Q_{i}}}{\sigma_{H,ideal}}$$

where $\sigma_{H,Q_{i}}$ signifies the maximum contact pressure computed for the examined quality grade $Q_{i}$, and $\sigma_{H,ideal}$ represents the maximum contact pressure calculated for the gear pair with the idealized geometry. The influences of elastic deformations and the consequent deflection of teeth under applied loads were incorporated by analyzing a range of feasible loads. The normalized tangential load values were $F_{T}/b=\left[6.67;13.33;\;20;\;26.67\right]$, and intermediate values were interpolated linearly.

## 3. Results

### 3.1. Crystallinity Measurements

**Figure 10:**
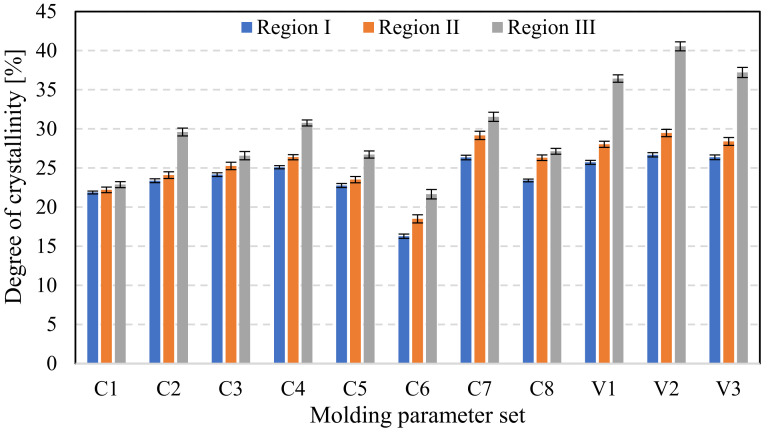
Measured degree of crystallinity in selected tooth regions (as presented in Figure 3). Crystallinity measurements were conducted on gear samples produced by various injection-molding parameter sets which are in detail presented in Table 3.

The results of the crystallinity study are shown in Figure 10. Crystallinity levels ranging from 16% ± 0.27% to 41% ± 0.6% were observed, showing a strong dependence on the process parameters and the location of measurement. The mold temperature and cooling time were found as the most influencing process parameters on the degree of crystallinity. Therefore, sets C4 and C7 exhibit the highest degree of crystallinity among the analyzed process parameters for the classical injection molding (C1 to C8). In all cases, the highest degree of crystallinity was measured in region III (tooth’s tip) in contrast to region I (tooth’s root), where the lowest levels were found. Melting temperature, packing pressure, and packing time were found not to have a significant effect on the degree of crystallinity. The reason for such behavior is that the tempering medium has less influence in the root region of the tooth. When employing the Variotherm technology (V1 to V3), increased levels of crystallinity were observed in all monitored tooth regions. The observed behavior was again correlated with the proximity of the tempering channel. Variotherm technology had the greatest effect on the area closest to the tempering channels. The greatest temperature variation during one cycle was in region III.

**Figure 11:**
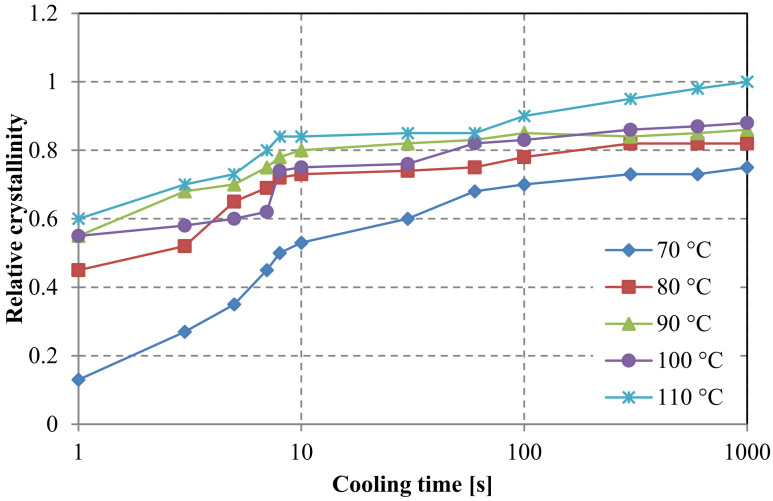
Dependence of crystallinity on the temperature and cooling time, curves generated for a neat resin.

For comparison reasons, crystallinity curves for a neat resin, generated by a Flash DSC method, are presented in Figure 11. Based on the presented measurements for the neat resin, preferred molding parameters can already be selected. It can be observed that a 90 °C mold temperature and cooling times between 10 and 20 s will result in a high degree of crystallinity.

### 3.2. Gear Quality Measurements

Geometric quality measurements for each gear, produced by different process parameters, are presented in Table 5. The accuracy grade was determined for the left and the right tooth flank. For each parameter, the worst result is included in the Table 5. This means that a certain percentage of gears had a better accuracy grade than the ones presented. Each parameter is defined by two values. The first value corresponds to the left flank and the second value to the right flank. Measurements were conducted in accordance with the DIN 3961/62 standard. On each gear, six teeth were measured. The overall resulting quality grade of produced gears ($Q_{R})$ ranged from Q10 to Q12, depending on the processing parameters.

### 3.3. The Influence of Lead Deviation on the Stress Condition in the Gear

**Figure 12:**
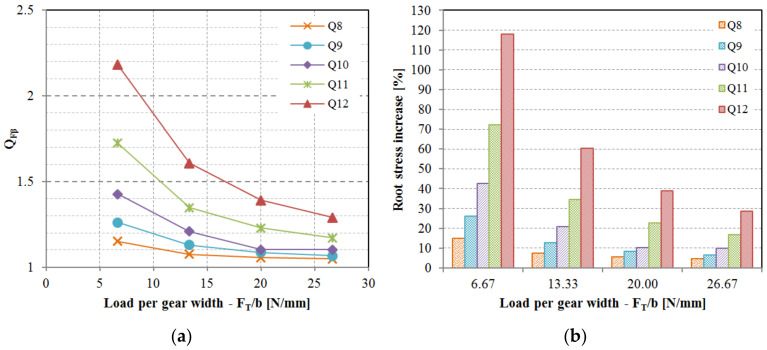
The effect of lead deviation on the root stress: (**a**) Factor $Q_{F\beta }$ considers the effect of lead deviation on the root stress increase, (**b**) Root stress increase depending on the lead quality grade and relative load on the tooth.

**Figure 13:**
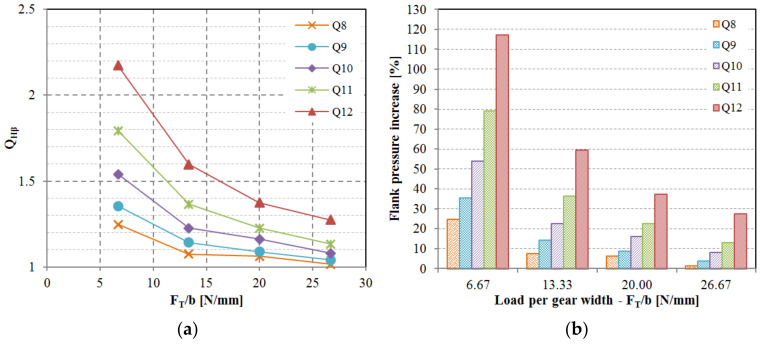
The effect of lead deviation on the flank pressure: (**a**) Factor $Q_{H\beta }$ considers the effect of lead deviation on the flank pressure increase, (**b**) flank pressure increase depending on the lead quality grade and relative load on the tooth.

The impact of lead deviation on root stress has been evaluated and can be integrated into the polymer gear design process by introducing novel factor $Q_{F\beta }$ in Equation (1). While the $K_{F\beta }$ factor is conceptually intended to account for lead deviations, it is important to note that several other potential deviations such as shaft misalignment and deflection have not yet been thoroughly explored in the context of polymer gears. As per the current state of knowledge, for practical purposes, $K_{F\beta }$ can be equated to $Q_{F\beta }$. Similarly, the standard factor $K_{H\beta }$, which includes lead deviation effects in conjunction with other factors not specifically addressed in this study, can also be approximated by $Q_{H\beta }$ as per Equation (2). The obtained values for the $Q_{F\beta }$ factor are displayed in Figure 12a, and the values for $Q_{H\beta }$ are illustrated in Figure 13a. It was observed that the influence of lead deviation diminishes with higher gear quality grades. The most significant impact of lead quality was noted for quality grades Q10, Q11, and Q12 at lower loads (Figure 12b and Figure 13b). As the load per gear width increases, teeth deflection becomes more pronounced and the effective tooth width expands, resulting in a reduction of the lead deviation effect. This pattern leads to a rapid decline in impact for Q12, Q11, and Q10 quality grades, whereas the effect is less pronounced for Q9 and Q8 quality grades. This observation highlights the potential areas for enhancing gear performance, indicating that improving the gear quality from Q12 to Q11 would have a more substantial stress reduction effect compared to enhancing the quality from Q9 to Q8. It is important to acknowledge that elevating gear quality from Q12 to Q11 or even Q10 can be realized through effective tool design and corrective iterations involving appropriate process parameters, while raising quality from Q9 to Q8 presents a significantly greater challenge.

### 3.4. The Influence of Pitch Deviation on the Stress Condition in the Gear

The standard factor *K_Fα_* addresses the influence of pitch deviation on the root stress in the gear, while the corresponding impact on flank pressure is accounted for by factor *K_Hα_*. Both factors incorporate the effects of potential deviations that may arise in gear pairs. However, this study exclusively delves into the ramifications of pitch deviation. Although the comprehensive evaluation of the pitch-deviation impact for polymer gear pairs has been scarce, for the sake of design calculations, $K_{F\alpha }$ can be aligned with $Q_{F\alpha }$ and *K_Hα_* with $Q_{H\alpha }$ according to Equations (1) and (2).

**Figure 14:**
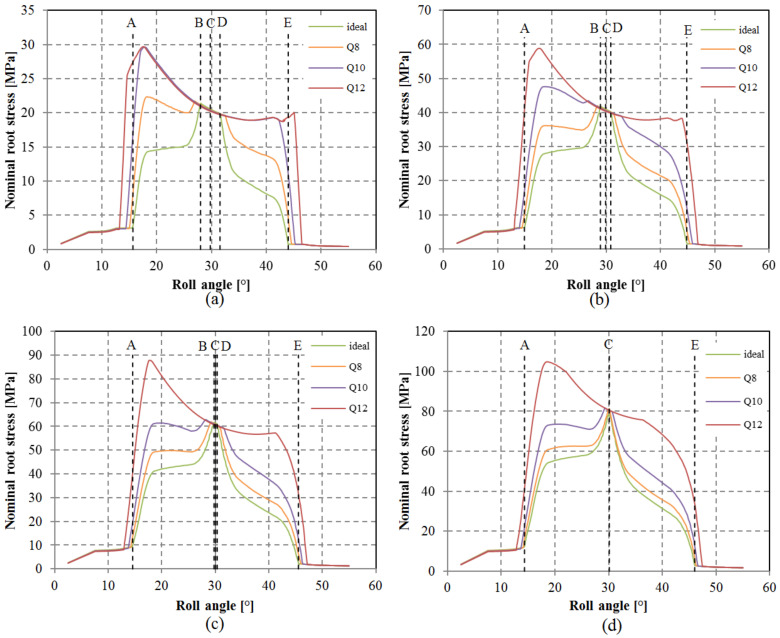
Evolution of root stress in the drive gear for analyzed loads and selected pitch quality grades: (**a**) 6.67 N/mm, (**b**) 11.33 N/mm, (**c**) 20 N/mm, (**d**) 26.67 N/mm.

**Figure 15:**
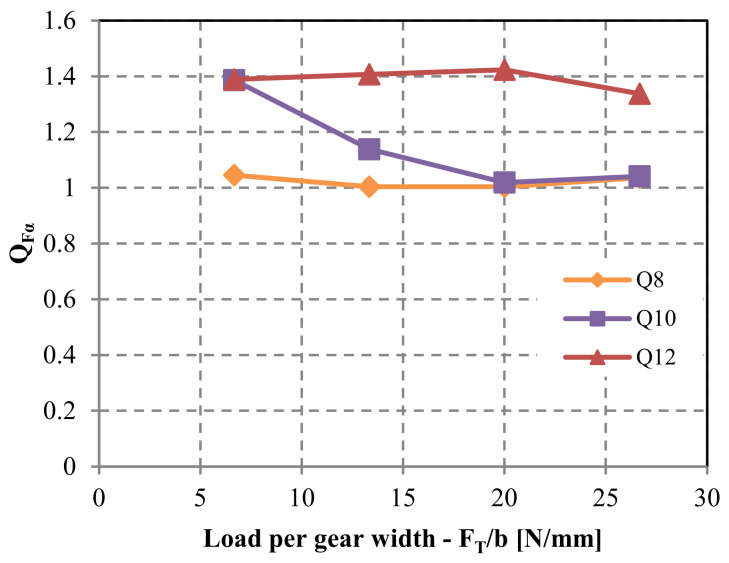
Factor $Q_{F\alpha }$ considers the effect of pitch deviation on the root stress for a POM/PA gear pair.

The ramifications of pitch deviation on root stress progression are depicted in Figure 14. In instances of lower-quality grades such as Q12 and Q10, coupled with low-to-medium loads, the computed root stress is most elevated during the initial stages of meshing. This suggests the presence of a solitary tooth engagement throughout the meshing cycle, rather than double-tooth contact. A comparison with the root stress evolution for an ideal geometry reveals that peak root stress should emerge when the gears mesh at the pinnacle of single-tooth contact. As the load increases, tooth deflection intensifies, leading to the establishment of double-tooth contact. The $Q_{F\alpha }$ factor, determined using Equation (6), is exhibited in Figure 15. The incorporation of tooth deflection effects is achieved through the analysis of varying normalized loads, encompassing cases of $F_{T}/b$ = [6.67; 13.33; 20; 26.67].

**Figure 16:**
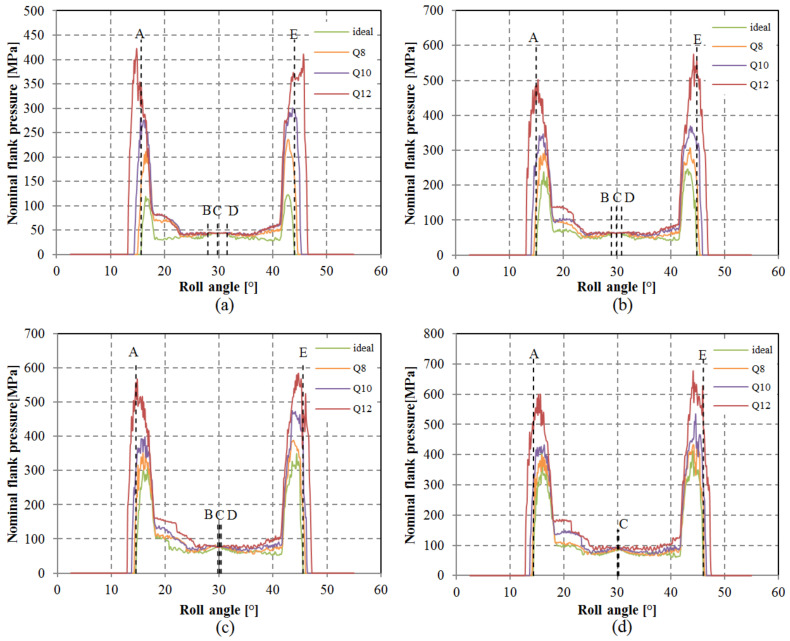
Calculated contact pressure for analyzed loads and selected pitch quality grades: (**a**) 6.67 N/mm, (**b**) 13.33 N/mm, (**c**) 20 N/mm, (**d**) 26.67 N/mm.

**Figure 17:**
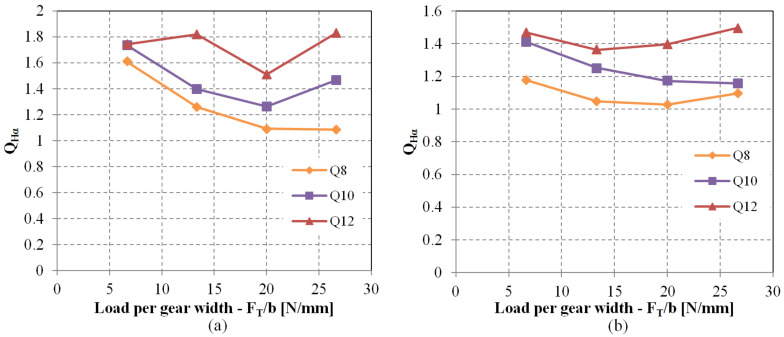
Factor $Q_{H\alpha }$ considers the effect of pitch deviation on the flank pressure for a POM/PA gear pair (pressure peaks at the start and end of meshing are excluded): (**a**) determined for meshing region between points A–B, (**b**) determined for the meshing region between points D and E.

Anticipating that pitch deviations could also influence contact pressure, this aspect was investigated as well. The evolution of contact pressure for the assessed load scenarios is outlined in Figure 16. Maximum contact pressure typically arises within the single-tooth contact region, spanning points B and D. Consequently, standard calculation methods extend contact pressure calculation to pitch point C or alternatively points B or D. Given that meshing points B, C, and D all reside within the single-tooth contact area, consistency prevails between calculated contact pressure for geometries featuring simulated deviations and an ideal geometry. However, variations were discernible at the commencement and conclusion of the meshing cycle, wherein lower gear-quality grades yielded elevated calculated contact pressure (Figure 17). These regions involve heightened sliding, which, in conjunction with augmented pressure, may lead to increased wear.

## 4. Limits of the Study

The study provides a framework that can be employed to evaluate the effects of a gear’s geometrical quality on the stress state in the gear. Several parameters still need to be studied and evaluated before gear quality effects can be accounted for by specified factors $K_{F\beta }$, $K_{F\alpha }$, $K_{H\beta }$ and $K_{H\alpha }$ in a standard calculation process, as provided by the VDI 2736 guideline.

The paper deals with a POM/POM gear pair combination, which is very often used in practical applications. By changing the material combination, for instance to Steel/POM, the load distribution between the meshing gears would be different, as the stiffness of the steel gear is much higher and only the teeth of the POM gear would comply. Studying the effect of the elastic modulus of both gears in the pair would be the next step to complete. Furthermore, different gear geometries, with different tooth foundation stiffnesses, would also produce different responses. The current study employs the gear geometry used in previous studies for which the molding tool was also available.

In relation to the elastic modulus, another parameter with a similar effect is the operating temperature. The material properties considered in the study, i.e., the elastic modulus, were as defined at the ambient temperature of 23 °C. With an increased temperature, the elastic modulus of polymer materials is significantly reduced. This would lead to increased teeth compliance, and the effects of geometric irregularities would be reduced. It is important to note, however, that with the increasing temperature, the fatigue strength of polymers also significantly drops. It can be speculated that the drop of fatigue strength would be higher than the decrease in stress concentration due to an improved load distribution.

The effects of lead and pitch deviation were evaluated; however, there are other quality parameters that might have an effect on the stress state. Studying the effects of other quality parameters, especially the runout, would be beneficial in order to gain a more comprehensive picture of the quality effects.

## 5. Conclusions

This study presents the effect of injection-molding parameters on the geometric quality of polymer gears. A correlation between the process parameters, geometrical quality, and degree of crystallinity was observed. The mold temperature and cooling time were found as the most influencing process parameters, where higher mold temperature and longer cooling cycles resulted in a more accurate gear geometry and a higher degree of crystallinity. In industrial processes, these two parameters tend to be as low as possible; hence, it is important to find the appropriate balance between the parameters and the resulting quality.

The study is expanded by studying the influence of gear quality on the resulting stress in a polymer gear during operation. The focus was on investigating the effect of lead and pitch deviations. The lead deviation exhibited its most significant impact within quality grades Q10 to Q12. Conversely, elevating the quality from Q10 to Q8 failed to yield a substantial enhancement in load distribution and the associated stress. Parallel observations applied to pitch deviation, wherein the most pronounced influence was evident while ascending the quality grade from Q12 to Q10.

This study revealed where the most effective changes can be achieved. The findings suggest that enhancing the gear quality from Q12 to Q10 can remarkably reduce stress levels (30% to 80% stress reduction, contingent upon the load), thereby leading to a consequential extension in gear lifespan. On the other hand, augmenting quality grades below Q10 offered a rather modest contribution to stress reduction (ranging between 5% and 20%, depending on the load). At this point, it also needs to be stressed that improving the gear quality from Q12 to Q11 or even Q10 can be achieved using proper tool design and corrective iterations with the right process parameters, while improving the quality from Q9 to Q8 is by far more challenging. These findings establish a valuable reference for gauging the extent to which enhancements in plastic gear quality are viable. To facilitate gear design calculations, novel quality factors were proposed.

## 6. Future Research

As noted by the limits of this study, there are several topics/parameters that still need to be researched/evaluated before gear quality effects can be included in the standard calculation process. To gain a more comprehensive insight, the effects of different material combinations, operating temperatures (elastic modulus decrease), running-in effects, gear geometries, and other gear quality parameters (e.g., runout) need to be further investigated.

## Figures and Tables

**Table 1:** Prescribed processing parameters as by the material’s manufacturer.

Parameter	Value
Melt temperature range [°C]	210–220
Melt temperature optimum [°C]	215
Mold temperature range [°C]	80–100
Mold temperature optimum [°C]	90
Drying time, dehumidified dryer [h]	2–4
Drying temperature [°C]	80
Processing moisture content [%]	<0.2
Hold pressure range [MPa]	−110

**Table 2:** Delrin 100 NC010 material properties, as defined by the material’s manufacturer datasheet.

Parameter	Standard	Unit	Value
Elastic modulus (23 °C)	ISO 527 [48]	MPa	2900
Yield stress (23 °C)	ISO 527	MPa	71
Melting temperature	ISO 11357 [49]		178 °C
Glass transition temperature	DIN53765 [50]		−35 °C
Density	ISO 1183 [51]		1.42 g/cm^3^

**Table 3:** Injection molding parameter sets selected for the production of test samples.

Nr.	Melt Temperature [°C]	Mold Temperature [°C]	Cooling Time [s]	Packing Pressure [MPa]	Packing Time [s]
Classical injection molding					
C1	210	90	30	80	5
C2	210	90	50	80	7
C3	210	130	30	120	5
C4	210	130	50	120	7
C5	220	90	50	100	5
C6	220	90	30	100	6
C7	220	130	50	80	5
C8	220	130	30	80	6
Variotherm					
V1	210	90	50	80	7
V2	215	110	30	80	6
V3	220	90	30	100	7

**Table 4:** Geometric parameters for the tested gear geometry.

Parameter	Value
Profile (ISO 53 [55])	A
Normal module [mm]	1
Number of teeth [/]	20
Face width [mm]	6
Reference diameter [mm]	20
Tip diameter [mm]	22
Profile shift [/]	0
Pressure angle [°]	20
Helix angle [°]	0

**Table 5:** Gear quality measurements conducted on gear samples produced by various injection-molding parameter sets which are in detail presented in Table 2.

Param.	C1	C2	C3	C4	C5	C6	C7	C8	V1	V2	V3
$F_{\alpha }$	9/9	9/9	12/12	10/10	8/8	8/9	11/11	12/12	8/8	11/11	11/11
$f_{f\alpha }$	7/6	9/9	9/8	7/6	8/8	10/9	8/8	8/9	6/5	7/7	6/7
$f_{H\alpha }$	10/10	9/9	12/12	11/11	9/8	9/10	12/12	12/12	8/9	12/12	12/12
$F_{\beta }$	7/7	8/8	9/8	11/11	8/8	9/9	8/8	9/9	6/6	5/7	6/6
$f_{f\beta }$	4/6	7/8	6/4	8/8	7/7	9/10	6/6	6/7	2/3	4/6	5/5
$f_{H\beta }$	8/8	8/9	10/9	12/12	9/9	9/9	9/9	10/10	7/7	7/8	7/6
$f_{p}$	5/6	7/8	7/6	10/10	7/7	10/10	6/5	7/7	6/6	8/8	8/8
$F_{p}$	8/8	11/11	10/9	12/12	10/10	9/9	7/7	10/10	9/10	11/11	11/11
$F_{R}$	9	12	11	12	10	12	9	11	10	12	12
$Q_{R}$	10	12	12	12	10	12	12	12	10	12	12

## Data Availability

The data presented in this study are available on request from the corresponding author.

## Nomenclature

b	mm	face width
$d_{i}$	mm	reference diameter
$d_{ai}$	mm	tip diameter
$d_{fi}$	mm	root diameter
$F_{T}$	N	tangential load
$m_{n}$	mm	normal module
$\sigma_{F}$	MPa	root stress
$K_{A}$	-	application factor
$K_{V}$	-	dynamic factor
$K_{F\beta }$	-	face load factor for tooth-root stress
$K_{F\alpha }$	-	transverse load factor for tooth-root stress
$K_{F}$	-	ctor for tooth-root load
$Y_{Fa}$	-	form factor
$Y_{Sa}$	-	stress correction factor (notch effect)
$Y_{\varepsilon }$	-	contact-ratio factor for root stress
$Y_{\beta }$	-	helix-angle factor for root stress
$\sigma_{H}$	MPa	flank pressure
$K_{H\beta }$	-	face load factor for flank pressure
$K_{H\alpha }$	-	transverse load factor for flank pressure
$Z_{E}$	-	elasticity factor
$Z_{H}$	-	zone factor
$Z_{\varepsilon }$	-	contact-ratio factor for flank pressure
$Z_{\beta }$	-	helix-angle factor for flank pressure
*Q*	-	quality grade
$Q_{H\beta }$	-	effect of lead deviation on the contact pressure
$\sigma_{H,Q_{i}}$	-	contact pressure calculated for the analyzed gear-quality grade
$\sigma_{H,ideal}$	-	contact pressure calculated for the theoretical gear geometry
$Q_{F\beta }$	-	effect of lead deviation on the root stress
$\sigma_{F,Q_{i}}$	-	root stress calculated for the analyzed gear-quality grade
$\sigma_{F,ideal}$	-	root stress calculated for the theoretical gear geometry
$Q_{F\alpha }$	-	effect of pitch deviation on the root stress
$Q_{H\alpha }$	-	effect of pitch deviation on the contact pressure
A_i_	-	initial point of tooth contact
B	-	lowest point of single-tooth contact (LPSTC) for the drive gear and the highest point of single tooth contact (HPSTC) for the driven gear
C	-	pitch point (kinematic point)
D	-	highest point of single-tooth contact (HPSTC) for the drive gear and the lowest point of single tooth contact (LPSTC) for the driven gear
E_i_	-	end point of tooth contact
$F_{\alpha }$	µm	total profile deviation
$f_{f\alpha }$	µm	profile form deviation
$f_{H\alpha }$	µm	profile slope deviation
$F_{\beta }$	µm	total helix deviation
$f_{f\beta }$	µm	helix form deviation
$f_{H\beta }$	µm	helix slope deviation
$f_{p}$	µm	single pitch deviation
$F_{p}$	µm	total cumulative pitch deviation
$F_{R}$	µm	runout
$Q_{R}$	-	overall quality grade of the measured gear
POM	-	poly-oxy-methylene
PA	-	polyamide
PEEK	-	poly-ether-ether-ketone
DSC	-	differential scanning calorimetry
PC	-	polycarbonate
